# Peer-to-Peer User Identity Verification Time Optimization in IoT Blockchain Network

**DOI:** 10.3390/s23042106

**Published:** 2023-02-13

**Authors:** Ammar Riadh Kairaldeen, Nor Fadzilah Abdullah, Asma Abu-Samah, Rosdiadee Nordin

**Affiliations:** Department of Electrical, Electronic and Systems Engineering, Faculty of Engineering and Built Environment, Universiti Kebangsaan Malaysia, Bangi 43600, Selangor, Malaysia

**Keywords:** digital integrity, user integrity, P2P, blockchain, smart contract, encryption algorithms, hash functions, Internet of Things (IoT), privacy protection

## Abstract

Blockchain introduces challenges related to the reliability of user identity and identity management systems; this includes detecting unfalsified identities linked to IoT applications. This study focuses on optimizing user identity verification time by employing an efficient encryption algorithm for the user signature in a peer-to-peer decentralized IoT blockchain network. To achieve this, a user signature-based identity management framework is examined by using various encryption techniques and contrasting various hash functions built on top of the Modified Merkle Hash Tree (MMHT) data structure algorithm. The paper presents the execution of varying dataset sizes based on transactions between nodes to test the scalability of the proposed design for secure blockchain communication. The results show that the MMHT data structure algorithm using SHA3 and AES-128 encryption algorithm gives the lowest execution time, offering a minimum of 36% gain in time optimization compared to other algorithms. This work shows that using the AES-128 encryption algorithm with the MMHT algorithm and SHA3 hash function not only identifies malicious codes but also improves user integrity check performance in a blockchain network, while ensuring network scalability. Therefore, this study presents the performance evaluation of a blockchain network considering its distinct types, properties, components, and algorithms’ taxonomy.

## 1. Introduction

The Internet of Things (IoT) is a technology-related concept in which devices which are used daily, including appliances, watches, etc., are connected to the Internet. The interconnection of IoT services is considered the central enabling technology for smart cities [1], which will revolutionize the way we conduct and manage business, critical infrastructure, healthcare, education, and entertainment in a secure and protected manner. As an essential application of IoT, a smart building (SB) automation system aims to incorporate equipment with sensors, actuators, and control devices to achieve operational efficiency and reliability, while significantly reducing operating costs. IoT devices’ lack of computational resources makes them unsuitable for intensive operations or large storage. This motivates the use of blockchain for IoT device management.

A blockchain is a distributed database of verifiable records containing transactions shared among participating parties and verified through consensus, where cryptographic hashes link the records within. In a heterogeneous blockchain network, the network must be identified and the identity allocated to different IoT nodes and individual users [2]. Digital identity, which is used to develop all the protocols related to security mechanisms, is one of the core concepts within security. Meanwhile, identity and access management (IAM) systems are useful for managing identity information with the help of operations set, such as register, revoke, look-up, and update functions. The IAM system holds various challenges. However, one of the main challenges is that IAM within IoT recognizes unfalsified identities attached to IoT appliances as a source of truth for user authentication and abnormal behaviour detection [1,3]. A recent review of security issues, challenges, and recommendations for blockchain technology has been presented in [4].

When managing IoT identities, initially, there is a need to recognize IoT devices and then allocate them to different identities available across the domain of the IoT, enforce security policies, and control their attitude or behaviour; all with the help of authentication and access control mechanisms [5]. For this reason, an identity verification framework based on blockchain technology could be utilized, which is one of the user-centric approaches toward managing the identities of IoT and facilitating their monitoring. In particular, blockchain is used to maintain all owners’ identities. However, identities associated with things are interrelated with the owner’s digital signature through the owner’s private key. The blockchain-based framework involves a methodology to filter, characterize, and monitor the appliances to extract digital signatures from the digital characteristics of the device [6]. Digital signatures based on identities and timestamps give blockchain an option for protecting, proving, and complying with rules, and auditing non-repudiation in data-intensive applications and ecosystems [7].

Thus, to make the smart home blockchain network more secure, non-interactive zero-knowledge proofs are considered a major building block, depicting the statement’s validity without disclosing any significant information. The zero-knowledge proof (ZKP) is one of the cryptographic techniques that demonstrates how a prover can confirm any particular statement without giving the verifier any vital information or disclosing information related to the witness. Apart from blockchain, the zero-knowledge protocol is an essential and versatile algorithm used for several privacy-oriented applications such as ethical behaviour and authentication systems [8].

Therefore, this article has the following significant contributions:

1. Identity management (IdM) system design based on a blockchain with a specific criterion to ensure user integrity and system performance.
2. Comparison of verification time with different user signature encryption algorithms using realistic datasets.
3. Selection of the optimum identity claim between encryption and hashing algorithms by considering network scalability and performance.

The rest of this paper is organized as follows. In Section 2, we present a brief background on integrity management, monitoring, and logging to highlight the identified authentication and key management system, relevant encryption algorithms, and streaming techniques that help to achieve this work. Section 3 describes the proposed architecture used in the blockchain for the IoT network and the process flowchart. Section 4 presents the implementation of the proposed user identity validation algorithm to analyze the execution time and the scalability of the network’s performance. Finally, in Section 5, we conclude with a detailed discussion of different issues involved in the proposed algorithms and the proposition to improve them.

## 2. Related Work

With the advancement of blockchain technology, many recent works have included discussions on blockchain security and privacy, such as the essential principles of blockchain identity related to data security and management [9] and the importance of blockchain in providing security and privacy to IoT devices [10,11]. However, the IoT centralized authentication system is not coherent with blockchain architecture [12], as a central server manages and controls device communication and provides the required identification and authentication. The handling of data security by the central authority (CA) eliminates the main essence of the blockchain concept.

An effective way to mitigate attacks on the intelligent IoT ecosystem is a decentralized architecture in which no central controlling authority exists. A centralized system needs to avoid even single-point failure and is vulnerable to common and routine cyber-attacks [13,14]. Instead, a consensus protocol is used to validate a transaction [10] through a decentralized identity management system that provides and manages unique identities [12]. This concept allows participants with permission access to view the same data simultaneously in a distributed network [13]. In this case, transactions and data are recorded identically in multiple locations. With blockchain, each node acts independently while connected to the rest of the network.

### 2.1. Identity and Access Management

Identity and Access Management (IAM) is the technology and policies framework that ensures authorized individuals within a firm possess suitable access to the network’s resources [14]. IAM provides three main components, namely, (i) identity management, (ii) access control, and (iii) monitoring and logging. With the help of this system, the firms’ resources are provided access control, which also monitors users’ activity. IAM offers the means for managing user authorizations based on their role in the company. It is regarded as the association of access control and identity management, which fulfill two primary goals: the orchestration and attribution of a digital identity to users (i.e., developer, admin, operator), service (i.e., database, application, web service), resource (i.e., computing power, data), or device (i.e., heavy machinery, sensor, RFID chips), together with authorization and authentication of such identities. The IAM lifecycle consists of permissions, authentication, self-service, provisioning, de-provisioning, and authorization. These are essential for secure machine-to-machine communication, especially in an IoT system [15].

IoT blockchain network hardware’s main components are sensors/devices, gateways, and network devices. Moreover, IoT architecture has management service and application layers, and each component configuration will depend on the application. Many limitations come with the sensor nodes, like low bandwidth, short communication range, and limited CPU processing power, memory, and energy [16]. An IoT gateway is a central hub that lets data flow in both directions between IoT devices and sensors on one side and cloud/server computing and data processing on the other.

According to [17], IoT devices must be uniquely recognizable to establish trust and prevent data corruption and spoofing. Permission configuration is a crucial IAM component, in which every actor should possess a set of actions that rely on their individual identities. For defining access control, different methods are utilized, such as attribute-based access control (ABAssC) or role-based access control (RBAC). The implementation of IAM for the Ethereum blockchain is presented in [18], in which the functions are performed with the facilitation of a smart contract for robust backup and monitoring functionality. With regard to smart contracts, the access control and identity are managed directly on the blockchain, and there is no need for any intermediary. The mechanism of access control utilized within the contribution is ABAC. A lightweight peer is hosted by the IoT gateways or devices to manage the communication between the blockchain and the smart system.

The IAM’s authorization and authentication validation procedure needs high trust levels, which should be quantifiable and have meaning. Public key infrastructure (PKI) is viewed as a de facto standard to provide electronic trust in a centralized management system. The reliability of PKI on the appropriate utilization of a private and public key pair relies on being a trusted chain among certificate authorities (CAs). Currently, smart contracts and blockchain have been introduced as distributed ledger technology (DLT) extensions. This has changed various aspects of management, business models, and components of IAM to a great extent within distinct use cases, such as healthcare, smart cities, smart homes, telecommunication, and IoT [19,20]. The decentralization, non-repudiation, immutability, and traceability of both technologies have made them attractive features for identity and access management. 

Many security and privacy threats are possible, especially in smart homes, and these need to be controlled by solving authorization issues and ensuring authorized users do not access sensitive resources. Hence, identity and access management offer a practical authorization framework that could secure smart home devices.

Access control is an important technique to address the problems of smart homes’ security, access, and privacy violation. It aims to ensure that only authorized users, services, and data can access the resources of the house [21,22] The system is protected by access control which limits legitimate users’ access according to their privileges and safeguards the privacy of other authorized users.

Meanwhile, identity management (IdM) is described as individual identity management along with maintaining privileges, authorization, roles, and authentication in an organization or within the boundaries. With identity management, all users are enabled by a distributed ledger network to obtain a similar truth source relating to the authentication or validity of the credentials, and for whom the data validity is attested inside those credentials, without disclosing actual data. The IdM offers various technologies and tools to decision-makers to control users’ access to critical information in a firm [23]. The primary functionality associated with the identity management system is to enhance productivity and security, which involves user creation or deletion, unlocking or locking users, and revoking and granting access. 

In IdM, the applied owner identity management procedure is dependent upon enabling the given features in the blockchain [1]:

- Any appliance owner can create digital identities as blockchain transactions without depending on third-party authorities.
- All digital identities are present worldwide and are accessible to check identity legitimacy.
- A scalable identity management approach based on a peer-to-peer network eliminates minor points of failure by removing its reliance on centralized servers and avoiding censorships.
- Grounded upon private/public keys, which are generated from the hierarchical deterministic of a wallet and hence can be applied to all entities of IoT irrespective of their heterogeneity.

However, it is noted that most organizations’ identity management systems are outdated and weak [8]. The identities need to be not only verifiable and portable but also secure and private. The utilization of blockchain technology in identity management has provided security and decentralized solutions, which have put users in control again due to the use of a distributed trust model. The utilization of blockchain identity management systems has removed the intermediaries making them more secure and reliable for users.

Monitoring and logging are essential for maintaining IAM systems’ performance, reliability, and availability. With blockchain collaboration, IAM offers log and monitoring solutions for users, which could help prevent the possibility of integrity violation and data loss. The study of [20] discusses one of the authentication methods in which blockchain utilization takes place as an authentication log storage. Within such a solution, the user’s access to 5G (fifth generation) networks is executed through the public key. If the validation of the user is successful, the network can be accessed by the device, and the login data can be safely stored inside the blockchain. Another platform is the decentralized runtime access monitoring system (DRAMS) [20], in which blockchain is utilized for the management of logs within the procedure of access control. DRAMS relies on smart contracts for storing records and implementing a policy analyzer to evaluate whether the decision relating to access is appropriate as per the available policies’ semantics. Moreover, a monitoring and backup functionality for smart homes has been proposed in [24] based on blockchain technology. Such a system ensures that an overall log of the encountered issues and transactions always remains within the blockchain.

### 2.2. User Authentication

System security and data validity are ensured with the help of a public and immutable blockchain ledger, which is considered the foundation of self-sovereign digital identity. In the authentication system of a blockchain, the owners could utilize the private key to differentiate themselves. As every user has their own key that cannot be used by other parties, the overall network is more private and secure.

Authors in [25] suggest that the first distributed public key infrastructure (PKI) system is based on blockchain technology linked to public user identities with a public key certificate via a public ledger record. This produces a decentralized PKI construction, enabling users to query the certificate’s issuance procedure. Moreover, it has been observed that user integrity authorization and authentication mechanisms are essential to secure IoT applications. Thus, with the utilization of blockchain technology, the security and privacy threats of users’ data are easily mitigated due to its efficient protocols and systems.

### 2.3. Key Management

A key exchange or key distribution protocol is needed before symmetric or asymmetric encryption can be adopted in the blockchain. However, the key exchange protocol is vulnerable to man-in-the-middle (MITM) attack because it does not authenticate the participants. This can be overcome by using digital signatures and public key certificates. This work considers symmetric and asymmetric encryption for key management and signature applications [26].

Symmetric key cryptography is also known as secret key cryptography. It is a kind of cryptography in which the sender and receiver can exchange information for end-to-end encryption and decryption. This means that the key is self-certified and only shared through a secure communication channel.

Conversely, asymmetric key cryptography, also known as public key cryptography, allows the sender to utilize a public key of the receiver mainly for encryption purposes, after which the receiver uses his private key to decrypt the message. One key aspect of conventional public key encryption is that it is less efficient for small mobile devices because it involves more mathematical functions. In [27], various asymmetric encryption algorithms are explored for symmetric key exchange purposes, namely the Rivest-Shamir-Adleman (RSA), Diffie-Hellman, ElGamal, and elliptic curve cryptography (ECC). Meanwhile, third-party public key authority and certificate authority (CA) can be used for public key distribution.

Another way of categorizing encryption is the block and stream ciphers. This refers to how the plaintext is processed [28]. Typically, the information is processed in chunks in a block cipher, while in a stream cipher, bit-by-bit information encryption is conducted. Stream and block ciphers are usually used with symmetric keys. This is for performance reasons because public key cryptography is much more expensive. Symmetric encryption algorithms are significantly faster than asymmetric algorithms [28]. This is mainly because less processing and computational power is required.

#### 2.3.1. Data Encryption Standard

Data Encryption Standard (DES) is a symmetric encryption algorithm that was standardized in 1977 which was developed by the National Institute of Standards and Technology (NIST). Typically, DES offers a standard method and mechanism to protect and safeguard any sensitive or uncategorized set of data. Typically, DES would include 64 bits as an input block within which 56-bit is the key, while 8 bits are usually used for odd parity checks. DES has a practical implementation in commercial, military domains, and public and state affairs [5]. However, in 1999 NIST announced that DES should only be used for legacy systems, and Triple DES was to be used instead because of concerns about brute-force attacks.

#### 2.3.2. Triple Data Encryption Standard

The Triple data encryption standard (3DES), is the upgraded version of the DES that was developed in 1998. Ideally, it works on the same principle as DES. However, it is three times slower than a regular DES system and requires higher power consumption. On the other hand, it is safer because the 3DES algorithm requires that the plaintext is encrypted using the first key, decrypted using the second key, and finally encrypted again using the third key before it is transmitted [28].

#### 2.3.3. Advanced Encryption Standard

Advanced Encryption Standard (AES) is a modern encryption standard formulated by NIST as another substitute for the DES algorithm and included in the ISO/IEC 18033-3 standard. AES is a symmetric block cipher proposed by Rijndael in 1998 and published by NIST in 2001. The cipher takes a plaintext block of 128 bits, while the key length can be three different versions known as AES-128, AES-192, and AES-256. The encryption and decryption number of rounds is based on the size of the key. A 128-bit key consists of 10 rounds, while a 192-bit key has 12 rounds, and a 256-bit key has 14 rounds. It is noteworthy that a cipher usually has a similar sequence of encryption and decryption algorithms. However, inverse transformation steps for AES occur during the decryption process [29].

#### 2.3.4. Blowfish

Blowfish is a symmetric block cipher 64-bit with a variable key size option ranging from 32 to 448 bits. It is therefore regarded as a fast encryption algorithm. Blowfish was also introduced in 1993 as a candidate to replace the DES encryption algorithm. However, it is still based on the Feistel cipher structure, similar to DES. Blowfish is a license-free block cipher that is accessible to all. Usually, the data encryption is performed through 16 rounds of the processing function to increase security. The complex key scheduling algorithm, and key-dependent permutation and substitution made it unpopular for modern applications [30].

#### 2.3.5. Twofish

Twofish is a symmetric block cipher containing a singular key for encryption and decryption introduced in 1999. It is an improvement to the Blowfish cipher by using a pre-computed substitution box. Twofish comprises a 128-bit plaintext block size and may accept a range of key lengths up to 256 bits. When implementing Twofish, ideally, three steps are used. The primary step consists of dividing the input bit into four different parts. The next step comprises XOR operation among the bit input with a key [31]. The final step includes processing the input bits for 16 rounds through the Feistel network. One key theoretical feature of Twofish is that it is unbreakable.

#### 2.3.6. Rivest-Shamir-Adleman

Rivest-Shamir-Adleman (RSA) is an asymmetric encryption algorithm that comprises private and public keys. This cipher was introduced in 1977 for digital signatures or key exchange algorithms. Ideally, RSA includes variable-length keys and variable-length blocks of encryption. In the RSA, the message is encrypted by the sender, which is usually the cloud service provider. When this happens, the receiver, which is the cloud service consumer, decrypts the message by utilizing a public key that is further decrypted with the help of a suitable private key owned by a receiver [32].

#### 2.3.7. Elliptic Curve Cryptography

Elliptic curve cryptography (ECC) is one of the most recent asymmetric encryption algorithms founded based on the elliptic curve theory in 1985. ECC contains complicated algebraic and geometric equations that create a public key. Therefore, ECC has public key cryptography and may employ a private key for the decryption and generation of signatures. However, the public key is used when encryption and verification are needed for signatures. Typically, ECC is employed to enhance the encryption algorithm, including the ECC–Diffie-Hellman and ECC-DSA. Therefore, ECC minimizes computing power and battery resource consumption [33]. As a result, it is used in mobile device applications to offer a fast and efficient model of the secured cloud application.

### 2.4. Digital Signature

A cryptographic digital signature is used to provide user integrity to verify and prove the originating source of a transaction. An asymmetric encryption algorithm is incorporated into digital signature protocols such as RSA encryption. In symmetric encryption algorithms, a private or secret key can also be used to provide user authentication functionality. Therefore, private keys are essential in symmetric cryptography, asymmetric cryptography, and blockchain. Private keys should only be shared with the key’s generator or parties authorized to decrypt the data.

## 3. System Model

### 3.1. User Identity Architecture Components

**Figure 1:**
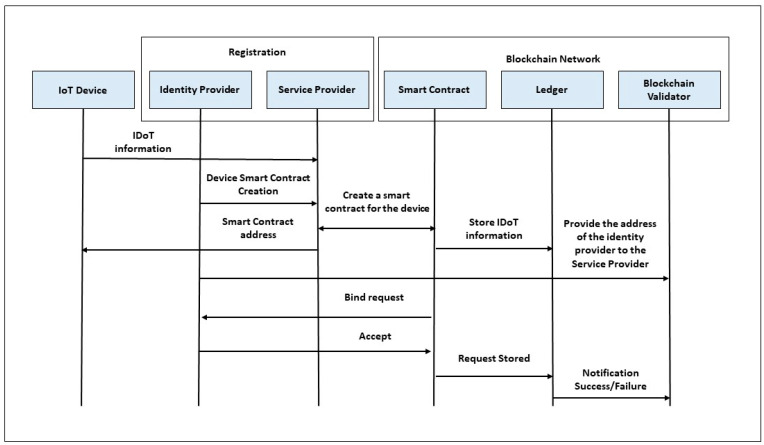
Proposed device registration process.

In this work, we propose an improved registration process [34] for the blockchain network with identity provider, as shown in Figure 1.

The device registration process in the blockchain network starts from the identity provider that enables this device or node to have the credentials in the blockchain network before creating the smart contract. Next, the service provider invokes the device information from the identity provider to authorize the associated privacy policies. Users obtain device information and privacy policies from the public variables of the identity provider. Therefore, the identity provider provides the addresses to the blockchain validator, who can then submit a request to bind the device to the device smart contract using the identity provider’s addresses, ensuring that the identity provider accepts the request and receives alerts. In addition, a combination of logging tools and real-time monitoring systems can be used to maintain optimal blockchain performance based on feedback from different components.

It is worthwhile to mention some limitations we didn’t consider in this work, like data maintenance using decryption of the original data blocks of the transaction before the hashing process.

In a decentralized permissioned blockchain network, users, or identity of things (IDoT), could be humans or smart devices interacting with each other or the sensors. All information is stored in the distributed ledger in the smart contract and accessed only by authorized nodes. Privacy and integrity are provided by several cryptographic algorithms. We proposed an identity management system using a symmetric or asymmetric algorithm and a digital signature for encryption and authentication.

**Figure 2:**
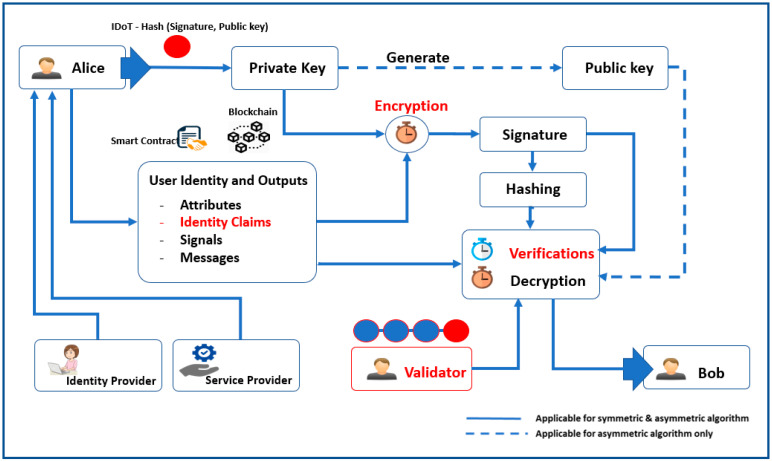
Architecture of proposed permissioned blockchain network identity management system.

Figure 2 shows the system design and workflow of our proposed blockchain network for IAM. The identity provider is responsible for permitting the participants, such as Alice, Bob, and a validator in our scenario to the network. Moreover, it emits identity claims about the network users. The service provider manages the permission to use the network. Meanwhile, the smart contract is the central core of the blockchain network in which all participants (i.e., Alice, Bob, validators, and any other node in the network) can immediately ascertain the outcome of the IAM procedure, without any intermediary’s involvement or time loss. The signature is created using an encryption method utilizing the private key, and the signature with hashing is used to verify the user’s identity in the validation process. In addition to handling the identity verification process, smart contracts also guarantee network transactions. Using one of the verification algorithms, the validator’s role is to ensure user integrity in case of falsified identity claims.

### 3.2. Data Structure and Hashing Using Merkle Hash Tree

**Figure 3:**
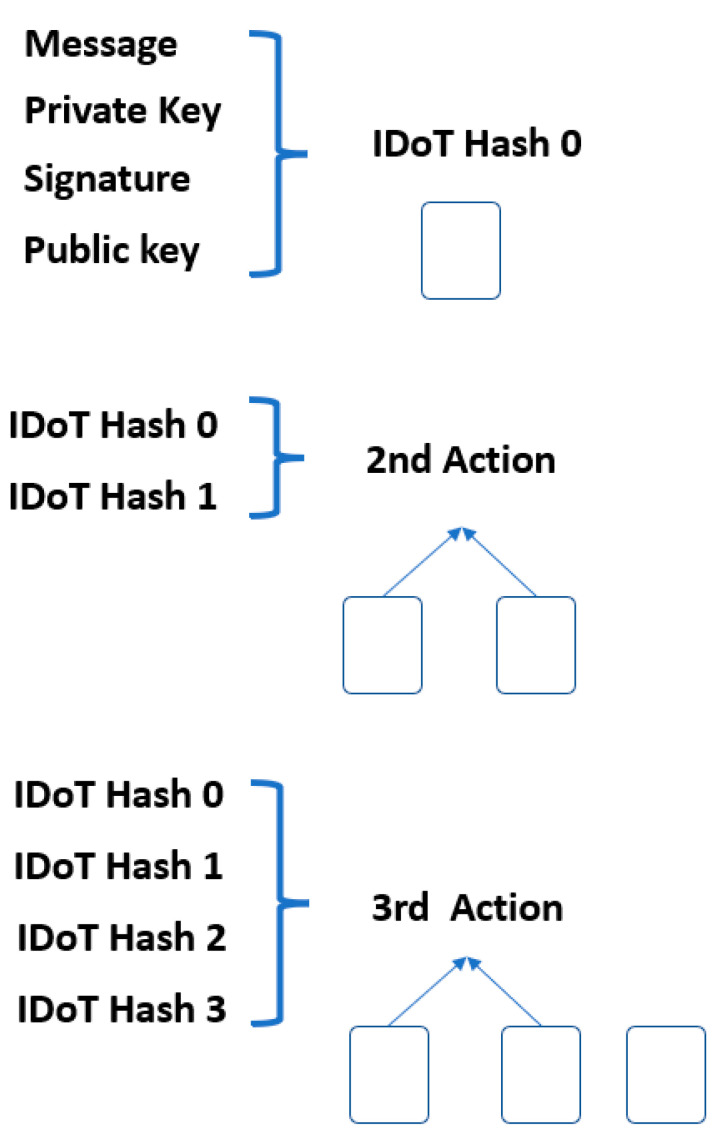
Transaction structure.

**Figure 4:**
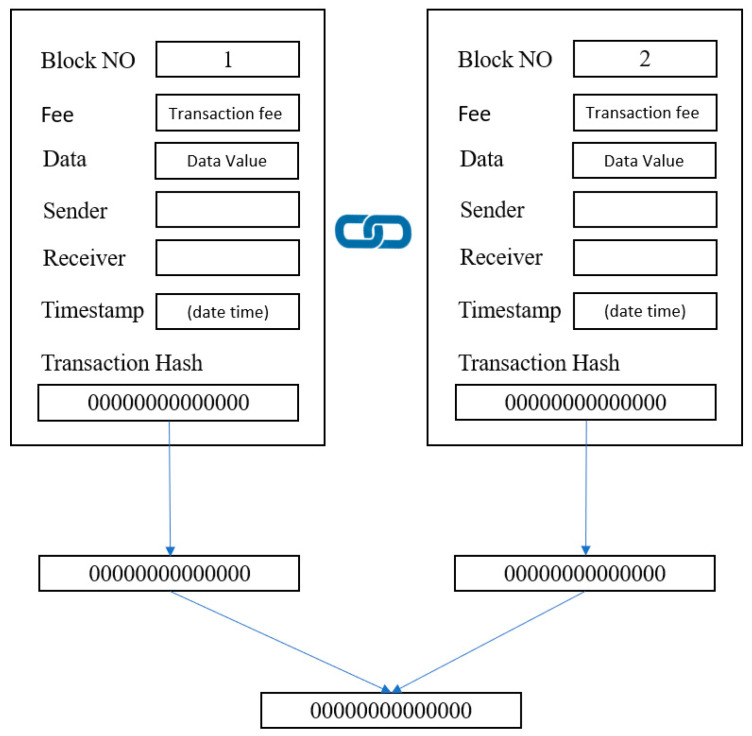
Blocks structure.

The transaction values were hashed into the transactions chain until the final transaction value was obtained. In a blockchain, the Merkle hash tree (MHT) algorithm is used to hash the data block and any transaction action added to the structure, as illustrated in Figure 3. Each block connects to the next block and block data structure and is shown in Figure 4.

**Figure 5:**
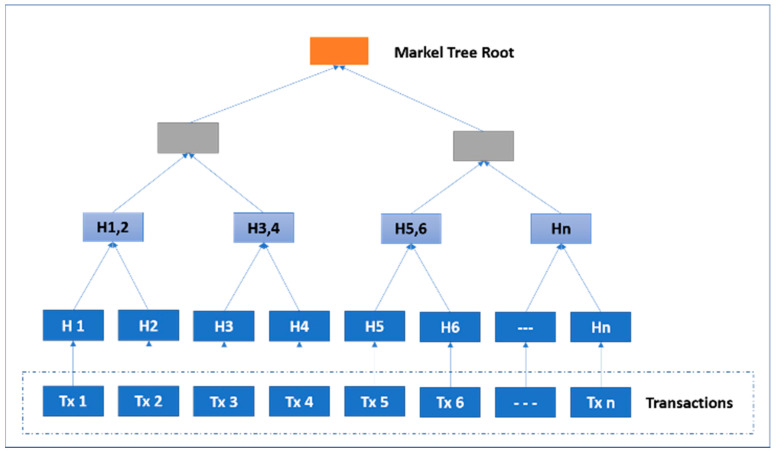
Merkle hash tree (MHT).

**Figure 6:**
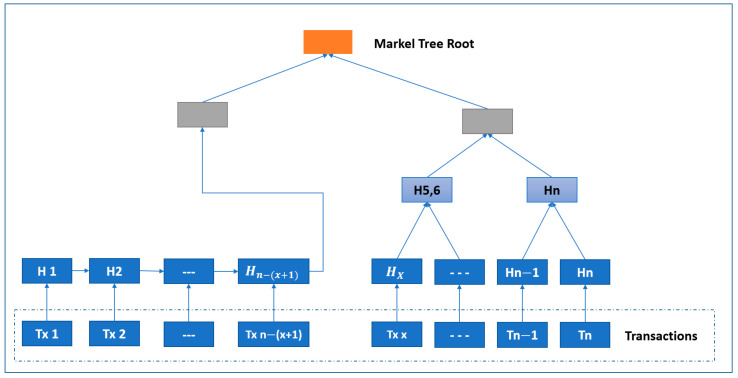
Modified Merkle hash tree (MMHT).

In this work, we compare the conventional MHT with our proposed modified Merkle hash tree (MMHT), as shown in Figure 5 and Figure 6. In general, the mathematical calculation of the MHT data structure is modified in MMHT to gain 30% of time optimization. This is achieved by separating the chain of transactions into concatenated hash transactions (CHT) and MHT and then combining them to obtain the final block of transactions [35], which is represented mathematically for *n* blocks in Equation (1):

(1) $$\begin{array}{ll} H_{0\to n}= & CHT\;H_{\left(0\to \left(n-\left(x+1\right)\right)\right)}\;||\;\;MHT\;H_{\left(\left(n-x\right)\to n\right)}\\ H_{0\to n}= & \left(H_{0\to 1}\;||\;H_{2\to 3}\;\left|\left|\ldots \;\right|\right|\;\;H_{\left(n\to x-2\right)-\left(n\to x-1\right)}\right)\\ & ||\;\;\left(H_{\left(n\to x\right)-\left(n\to x+1\right)}\;\left|\left|\ldots \;\right|\right|\;H_{n}\right) \end{array}$$

## 4. Results and Discussions

The proposed system aims to provide more secure and faster execution of identity management in the blockchain. Therefore, two metrics were used in this study to evaluate the performance. The first metric is the user identity verification time, while the second is the efficiency of the encryption algorithm.

The technical comparisons between the results are based on the key size of each algorithm and the CPU processing speed for data encryption and hashing, which is based on the efficiency of hardware and software implementation and the amount of memory used to hold the data in the encryption process. The specifications of the local server representing the validator node in the blockchain are summarized in Table 1.

Different encryption algorithms were used in our proposed system model to compare the findings and assess the efficiency. Hence, this helped identify the most efficient consensus algorithm for the blockchain network and the ability to enhance identity security and integrity. Some modifications were also made to the data structure algorithm to increase its performance and overcome its complexity. Furthermore, three different transaction sizes (30, 3k, and 30k) were tested to verify the network user’s integrity performance at various transaction scalability levels. The results are produced in two stages: user encryption and blockchain hashing.

### 4.1. Stage 1: Signature Algorithm

This work evaluates seven encryption algorithms (a combination of RSA with five hash functions, Triple DES and AES) to provide the signature functionality. The comparison of several algorithms has the purpose of identifying the most efficient encryption algorithm for user signature in a blockchain network and the ability to enhance data security and integrity.

(2) $$Sig=En\;(PK,\;(H_{0-1}\;||\;H_{2-3}\ldots \left|\right|H_{n}))$$

Signatures (*Sig*) are generated by encrypting the private key (*PK*), and the final hash of the transactions data $((H_{0-1}\;||\;H_{2-3}\ldots \left|\right|H_{n}))$, as represented in Equation (2).

We also compared the results with other works [34,35] to provide a better perspective on the performance of the compared methods. In [36], only systematic key cryptographic techniques were considered to secure cloud computing in the same encryption process. Moreover, the small transactions size was observed in [36,37]. In this paper, we consider both symmetric and asymmetric algorithms, as well as a varying number of transactions, to represent the scalability of the blockchain network. Specifically, the findings of the signature generation execution time validation for three different transactions size were considered. The evaluation was performed based on ten average simulation runs with a confidence interval of 90% to ensure the results’ high accuracy and credibility.

**Figure 7:**
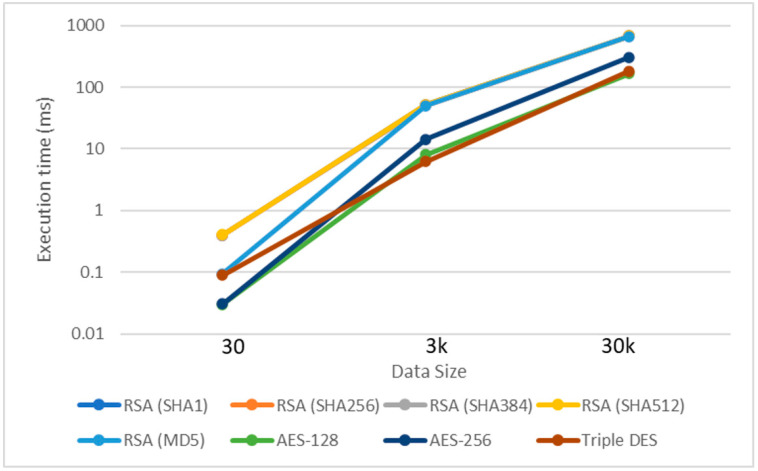
Signature algorithm execution time.

**Figure 8:**
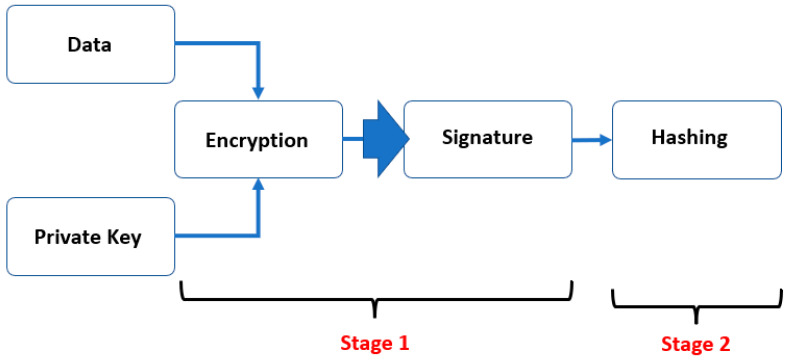
The encryption algorithm (Stage 1) and hashing algorithm (Stage 2).

The results of 30, 3k, and 30k transactions shown in Table 2 and Figure 7, record the execution time in milliseconds (ms). The table is categorized into symmetric and asymmetric cryptographic keys. Meanwhile, Figure 8 compares the execution time on a logarithmic scale. It can be seen that symmetric encryption has a higher execution time compared to asymmetric encryption. From the public key group, the RSA algorithm using the MD5 hash function has the best execution time, significantly different from the other algorithms for the 30 transactions dataset.

However, symmetric encryptions generally have a significantly better execution time than asymmetric algorithm execution. It can be seen that the AES-128 algorithm has the lowest execution time from the smallest 30 transactions up to the largest 30k transactions. This proves that the AES-128 is a scalable algorithm that gives the best execution time in the blockchain network.

### 4.2. Stage 2: Blockchain Hashing Algorithm

From the MHT and MMHT design architecture shown in Figure 5 and Figure 6, the blockchain network works by adding hashing procedure to the distributed chain to validate the transactions. As a result, the total execution time is the time taken to complete the first stage *En* (encryption) and the second stage *H* (hashing) operations using either MHT or MMHT, as shown in Equation (3) and Figure 8.

(3) $$Execution\;time=Stage\;1\;\left(En\right)+Stage\;2\;\left(H\right)$$

**Figure 9:**
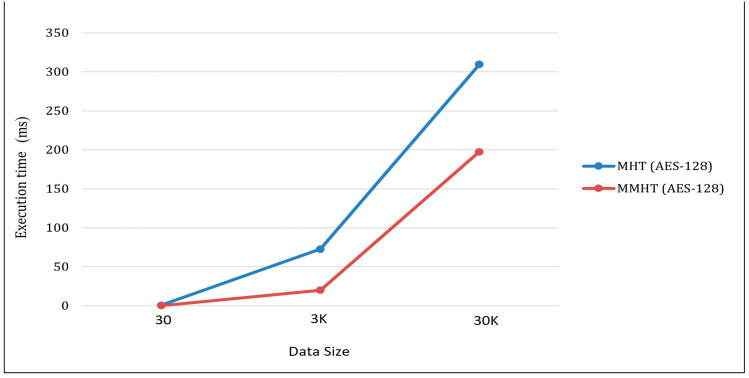
Comparison of hashing execution time (MHT & MMHT) using AES-128 symmetric cipher algorithm with three different dataset sizes.

Table 3 shows the results from large-scale 30k transactions using MHT, while Table 4 shows the results using MMHT. This is an extension to our previous work in [34] which studied various hash functions for MHT and MMHT blockchain networks, but did not include user integrity when using the signature. For the asymmetric encryption algorithm, RSA (MD5) integrated with SHA384 gives the best performance for the MHT algorithm as seen in Table 3, while RSA (MD5) integrated with SHA3 gives the most time optimum using MMHT. On the other hand, for the symmetric algorithm, the integration of AES-128 in Stage 1 and SHA3 in Stage 2 gives the optimum execution time over the asymmetric algorithms for both MHT and MMHT algorithms. Note that AES-128 is faster than AES-256 in execution time because of the smaller key size, but AES-256 is more robust against a brute-force attack by requiring more quantum computing power and a massive number of years to break the algorithm. However, for a blockchain network, AES-128 is more optimal in security and execution time implementation. Therefore, we highlight the execution time of AES-128 for different transactions size, as shown in Figure 9.

## 5. Conclusions

This work proposed a blockchain system based on identity and service providers, encryption, structure hashing algorithms, and other decentralized permissioned blockchain components. User verification and encryption in a blockchain network combined with identity management systems for IoT provide high security against any possible identity threats. A practical design of identity signatures can be effectively used in decentralized IoT blockchain networks. The design and architecture of an identity management system with different criteria are utilized to ensure user integrity and system performance. Furthermore, encryption using various algorithms based on the Merkle hash tree algorithm in both traditional and modified versions was adopted for user integrity verification check, comparing 15 different hash functions to find the optimum hash function tested in the data structure algorithm. Encryption using a symmetric AES key algorithm showed a significantly lower execution time than the asymmetric key RSA algorithm. The results showed that the AES-128 encryption and MMHT algorithm has the best execution time contribution of 36% compared with other encryption algorithms and hash function groups.

## Figures and Tables

**Table 1:** Summary of local server specifications.

Component	Description
CPU	Intel(R) Core (TM) i7-8550U CPU @ 1.80GHz 1.99 GHz
RAM	16.0 GB Speed 2133 MHz
OS	Windows 10 Pro, version 20H2, 64-bit operating system, x64-based processor
Disk Type	SSD SAMSUNG MZVLB512HAJQ-000L7

**Table 2:** Comparison of signature algorithm execution time in milliseconds.

Stage 1 (*En*)		Transaction Size		
		30	3K	30K
Asymmetric Cipher Algorithm	RSA (SHA1)	0.40283	51.54745	675.93811
	RSA (SHA256)	0.39781	51.044531	670.90891
	RSA (SHA384)	0.39952	51.21718	672.63543
	RSA (SHA512)	0.40694	51.95904	680.05403
	RSA (MD5)	0.09548	50.56269	666.09051
Symmetric Cipher Algorithm	AES-128	0.03045	8.1072	167.2961
	AES-256	0.03086	14.35118	303.97543
	Triple DES	0.08975	6.28985	183.36211

**Table 3:** Comparison of integrated signature and MHT algorithm execution time using 30k transactions dataset.

Stage 1 (*En*) + Stage 2 (*H*)		Encryption + MHT (30k Transactions) (in ms)							
		Asymmetric Cipher Algorithm					Symmetric Cipher Algorithm		
		RSA (SHA1)	RSA (SHA256)	RSA (SHA384)	RSA (SHA512)	RSA (MD5)	AES-128	AES-256	Triple DES
MHT Execution Time for 30 transactions (ms)	SHA1	974.43600	969.40680	971.13333	978.55193	964.58840	423.47333	602.47333	481.86000
	SHA256	1095.63600	1090.60680	1092.33333	1099.75193	1085.78840	544.67333	723.67333	603.06000
	SHA384	917.13600	912.10680	913.83333	921.25193	259.50000	366.17333	545.17333	424.56000
	SHA512	912.93600	907.90680	909.63333	917.05193	903.08840	361.97333	540.97333	420.36000
	MD2	1035.63600	1030.60680	1032.33333	1039.75193	1025.78840	484.67333	663.67333	543.06000
	MD5	868.23600	863.20680	864.93333	872.35193	858.38840	317.27333	496.27333	375.66000
	SHA3	856.53600	851.50680	853.23333	860.65193	846.68840	309.57333	484.57333	363.96000
	RIPeMD160	1088.73600	1083.70680	1085.43333	1092.85193	1078.88840	537.77333	716.77333	596.16000
	RIPeMD128	858.33600	853.30680	855.03333	862.45193	848.48840	307.37333	486.37333	365.76000
	RIPeMD256	914.73600	909.70680	911.43333	918.85193	904.88840	363.77333	542.77333	422.16000
	RIPeMD320	1038.63600	1033.60680	1035.33333	1042.75193	1028.78840	487.67333	666.67333	546.06000
	Tiger	933.63600	928.60680	930.33333	937.75193	923.78840	382.67333	561.67333	441.06000
	Whirlpool	862.23600	857.20680	858.93333	866.35193	852.38840	311.27333	490.27333	369.66000
	Gost3411	1012.23600	1007.20680	1008.93333	1016.35193	1002.38840	461.27333	640.27333	519.66000
	Shake	862.23600	857.20680	858.93333	866.35193	852.38840	311.27333	490.27333	369.66000

**Table 4:** Comparison of integrated signature and MMHT algorithm execution time using 30k transactions dataset.

Stage 1 (*En*) + Stage 2 (*H*)		Encryptions + MMHT (30k Transactions) (in ms)							
		Asymmetric Cipher Algorithm					Symmetric Cipher Algorithm		
		RSA (SAH1)	RSA (SAH256)	RSA (SHA384)	RSA (SHA512)	RSA (MD5)	AES-128	AES-256	Triple DES
MMHT Execution Time for 30 transactions (ms)	SHA1	316.82192	316.82175	316.82181	316.82206	316.82159	315.3465	316.80952	316.80550
	SHA256	438.02192	438.02175	438.02181	438.02206	438.02159	436.5465	438.00952	438.00550
	SHA384	259.52192	259.52175	259.52181	259.52206	259.50000	258.0465	259.50952	259.50550
	SHA512	255.32192	255.32175	255.32181	255.32206	255.32159	253.8465	255.30952	255.30550
	MD2	378.02192	378.02175	378.02181	378.02206	378.02159	376.5465	378.00952	378.00550
	MD5	210.62192	210.62175	210.62181	210.62206	210.62159	209.1465	210.60952	210.60550
	SHA3	198.92192	198.92175	198.92181	198.92206	198.92159	197.4465	198.90952	198.90550
	RIPeMD160	431.12192	431.12175	431.12181	431.12206	431.12159	429.6465	431.10952	431.10550
	RIPeMD128	200.72192	200.72175	200.72181	200.72206	200.72159	199.2465	200.70952	200.70550
	RIPeMD256	257.12192	257.12175	257.12181	257.12206	257.12159	255.6465	257.10952	257.10550
	RIPeMD320	381.02192	381.02175	381.02181	381.02206	381.02159	379.5465	381.00952	381.00550
	Tiger	276.02192	276.02175	276.02181	276.02206	276.02159	274.5465	276.00952	276.00550
	Whirlpool	204.62192	204.62175	204.62181	204.62206	204.60000	203.1465	204.60952	204.60550
	Gost3411	354.62192	354.62175	354.62181	354.62206	354.62159	353.1465	354.60952	354.60550
	Shake	204.62192	204.62175	204.62181	204.62206	204.62159	203.1465	204.60952	204.60550

## Data Availability

Publicly available datasets were analyzed in this study. This data can be found here: https://doi.org/10.5281/zenodo.3557461.
